# Obesity and type-2 diabetes as inducers of premature cellular senescence and ageing

**DOI:** 10.1007/s10522-018-9763-7

**Published:** 2018-07-27

**Authors:** Dominick G. A. Burton, Richard G. A. Faragher

**Affiliations:** 1 Wolfson School of Manufacturing and Mechanical Engineering, Holywell Park, Centre for Biological Engineering, Loughborough University, Loughborough, LE11 3TU UK; 2 School of Pharmacy and Biomolecular Sciences, University of Brighton, Cockcroft Building, Moulsecoomb, Brighton, BN2 4GJ UK

**Keywords:** Diabetes, Obesity, Senescence, Ageing, ox-LDL, Glucose

## Abstract

Cellular senescence is now considered as a major mechanism in the development and progression of various diseases and this may include metabolic diseases such as obesity and type-2 diabetes. The presence of obesity and diabetes is a major risk factor in the development of additional health conditions, such as cardiovascular disease, kidney disease and cancer. Since senescent cells can drive disease development, obesity and diabetes can potentially create an environment that accelerates cell senescence within other tissues of the body. This can consequently manifest as age-related biological impairments and secondary diseases. Cell senescence in cell types linked with obesity and diabetes, namely adipocytes and pancreatic beta cells will be explored, followed by a discussion on the role of obesity and diabetes in accelerating ageing through induction of premature cell senescence mediated by high glucose levels and oxidised low-density lipoproteins. Particular emphasis will be placed on accelerated cell senescence in endothelial progenitor cells, endothelial cells and vascular smooth muscle cells with relation to cardiovascular disease and proximal tubular cells with relation to kidney disease. A summary of the potential strategies for therapeutically targeting senescent cells for improving health is also presented.

## Introduction

The senescent state is a stress response which ensures that cell damage is removed through activation of the immune system. During this response, often induced by persistent DNA damage, cells lose their ability to proliferate; ensuring cells do not become cancerous. In addition, senescent cells develop an altered secretome consisting of pro-inflammatory factors, growth factors and proteases. Thus, senescent cells appear to mimic a wound healing response. Components of the senescent secretome function in attracting immune cells for the elimination of these damaged cells, thereby promoting the restoration of tissue homeostasis (Burton and Faragher 2015). However, during biological ageing and disease processes, senescent cells can accumulate. It is suspected that an ageing immune system (immunosenescence) may contribute to the accumulation of senescent cells through failure to remove them.

Metabolic diseases are associated with a disruption in normal cell metabolism, the process of converting food to energy on a cellular level. Such diseases impact the capacity of the cell to undertake essential biochemical reactions that involve the transport or processing of proteins, carbohydrates and lipids. Both obesity and type 2 diabetes are known to alter cell metabolism (Singla et al. 2010). Diabetes is a global health problem, estimated to be affecting 422 million people worldwide in 2014 and predicted to become the seventh leading cause of death in 2030 (World Health Organisation). Therefore, understanding the mechanisms promoting metabolic diseases and the biological consequence of such diseases is essential for the development of novel therapeutics. Cellular senescence may be one mechanism contributing to obesity and diabetes. In addition, the presence of obesity and diabetes can promote secondary diseases such as cardiovascular disease (CVD) and kidney disease, potentially through induction of premature cell senescence in other tissues. As such, the mechanisms and processes by which obesity and diabetes promote premature senescence will be the focus of this review.

## Cellular senescence as a mechanism of ageing and disease

**Fig. 1:**
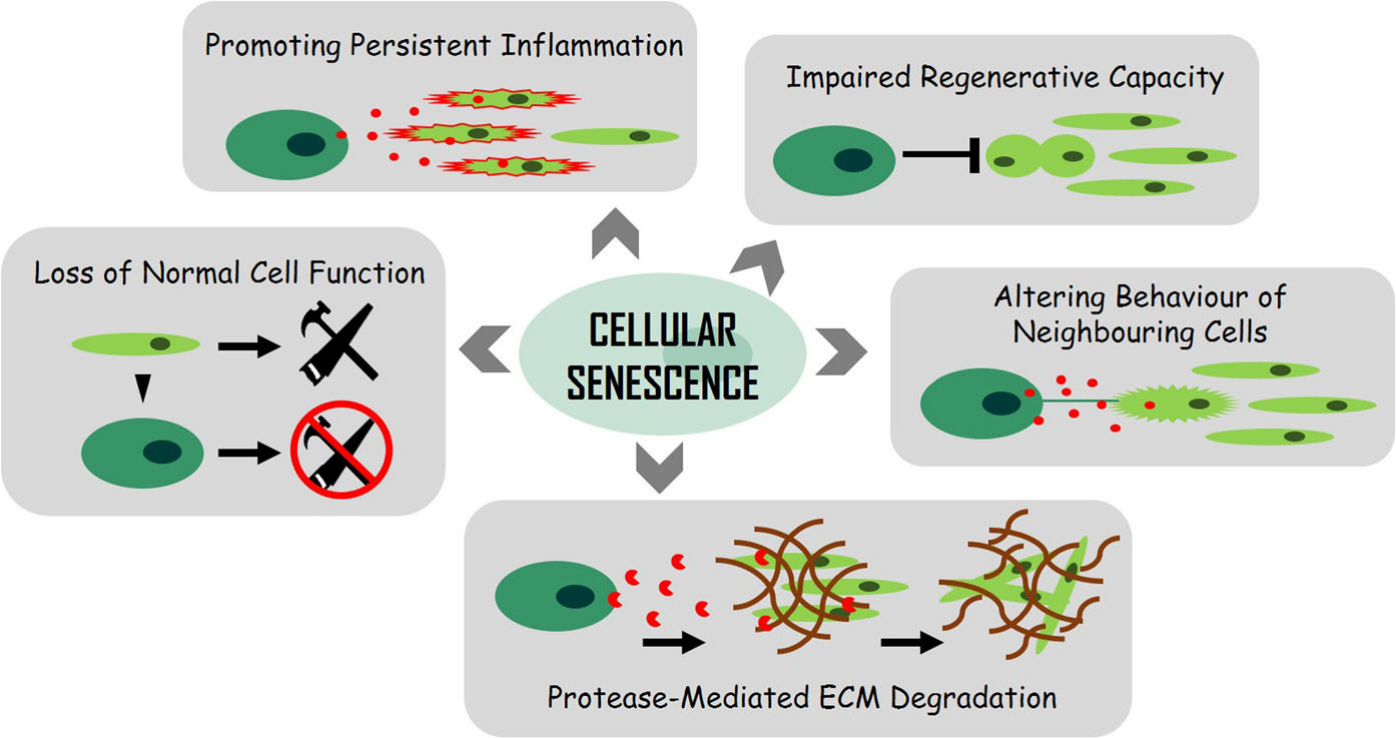
Mechanisms by which senescent cells promote ageing and age-related disease

Senescent cells have been associated with many age-related diseases and the subject has been extensively reviewed in recent years (Burton 2009; Childs et al. 2015; Muñoz-Espín and Serrano 2014; Ovadya and Krizhanovsky 2014; Sikora et al. 2014; van Deursen 2014) and so will not be explored here in depth. Instead, the underlying conceptual mechanisms as to how senescent cells could cause a decline in biological function underlying ageing and disease will be discussed (Fig. 1).

There are a number of mechanisms by which the senescent state can contribute to both natural ageing and accelerated ageing, both of which can manifest as age-related disease and as such, “ageing” and “age-related disease” can be considered as indistinguishable processes (reviewed in Faragher 2015). Natural ageing here is referred to as the gradual decline in biological function over the lifespan of an organism, whereas accelerated ageing likely involves the same processes but at a quicker rate due to additional stresses such as smoking, exposure to toxins, chemotherapy, high-fat diet and the presence of infectious disease. Accelerated ageing is more likely to impact certain tissues/organs rather than the organism as a whole. For example, chronic obstructive pulmonary disease (COPD) caused by smoking is probably the result of accelerated lung ageing induced by cigarette smoke (Ito and Barnes 2009; Mercado et al. 2015). The senescent state has the potential to induce biological dysfunction through their (1) inability to proliferate, (2) loss of normal cell function, (3) the secretion of pro-inflammatory factors, (4) altering the behaviour of neighbouring cells and (5) protease-mediated degradation of extracellular components (Fig. 1).

### Irreversible proliferative arrest

Cell turnover is essential for replacement of damaged/lost cells which occurs throughout the lifespan of an organism. As such, the presence of permanently arrested senescent cells would consequently reduce the number of cells capable of regenerating new tissue, more so if stem/progenitor cell senescence occurs. Such a decline in the regenerative capacity of cells would not only impact growth competent somatic tissues, but also post-mitotic tissues that rely on these growth competent cells to maintain their normal function.

### Loss of cell function

When a cell becomes senescent they can no longer undertake their original function effectively, potentially leading to a decline in overall tissue function. This may partly be due to drastic alterations in gene expression, some of which are senescent specific, some of which may be cell type specific. Furthermore, it has been speculated that senescent cells undergo promiscuous gene expression as a consequence of stochastic activation of genes due to continuous chromatin remodelling (reviewed in Burton and Faragher 2015). Examples of senescence-mediated functional cellular decline include (1) the inability of senescent vascular endothelial cells to generate nitric oxide required for regulating coronary vascular tone (Matsushita et al. 2001), (2) impaired insulin secretion by senescent pancreatic beta cells required for regulating glucose levels (Sone and Kagawa 2005), (3) reduced vascular tone due to the pro-calcificatory phenotype of senescent vascular smooth muscle cells (Burton et al. 2009; Nakano-Kurimoto et al. 2009) and (4) impaired adipogenesis due to a decline in adipogenic differentiation genes in senescent adipose progenitor cells (Mitterberger et al. 2014). Senescence research to date has primarily been focused on phenotypic similarities between different cell types rather than alterations, particularly functional abnormalities, which may be specific to certain senescent cell populations. An understanding of these senescent cell type specific responses will likely provide further insight with regard to their relationship to disease processes.

### Pro-inflammatory secretome

If senescent cells persist in tissues, continuous secretion of pro-inflammatory factors have the potential to induce tissue injury that ultimately manifests as disease. Persistent inflammation can cause tissue injury by a number of routes, including; inducing cell death, elevating ROS, persistent activation of immune cells and altering the tissue microenvironment (Mittal et al. 2014; Wallach et al. 2014). Some studies also suggest that inflammatory factors such as cytokines may also accelerate cellular senescence in neighbouring cells (Acosta et al. 2013). Due to the potential detrimental consequence of senescence-mediated inflammation, a number of studies have focused their efforts on identifying molecular pathways that could be targeted to inhibit this inflammatory response (Alimbetov et al. 2016; Laberge et al. 2012b; Xu et al. 2015b).

### Altering the behaviour of neighbouring cells

Pro-inflammatory factors in addition to other secretory components associated with cell senescence likely impact the behaviour/function of neighbouring cells via paracrine signalling. One of the more widely studied aspects of this senescence-mediated paracrine response is its ability to stimulate cell proliferation and invasion in premalignant and malignant cells (Krtolica et al. 2001; Laberge et al. 2012a) in addition to promoting age-related hyperplasia (Castro et al. 2003; Vital et al. 2014) and angiogenesis (Coppé et al. 2006). During normal wound healing, stimulated proliferation and angiogenesis by transient senescent cells is likely beneficial, but aberrant signalling via persistent senescent cells consequently stimulates unwarranted proliferation and cell behaviour associated with disease. Whilst direct transfer of proteins and cell organelles from senescent cells to NK cells appear to facilitate NK activation as mentioned previously (Biran et al. 2015), the same processes could potentially be hijacked by pre-cancerous and cancer cells to promote cancer development/progression through obtaining valuable cell resources (Biran and Krizhanovsky 2015). Senescent cells could also potentially alter the behaviour of neighbouring cells via secretion of extracellular vesicles such as exosomes that may contain regulatory components such as microRNAs (Xu and Tahara 2013). Future research will likely uncover additional processes whereby senescent cells can communicate and influence neighbouring cells.

### Degradation of extracellular proteins

The final process by which senescent cells could promote age-related disease is via the degradation of extracellular proteins. The extracellular matrix (ECM) is essential for normal intercellular communication required to maintain optimal tissue homeostasis and for providing structural and biochemical support to cells (Cox and Erler 2011). As such, ECM degradation by proteases can potentially disrupt normal tissue function that manifests as disease. Senescent cells often secrete proteases, particularly matrix metalloproteases (MMPs) as part of their wound healing response and as such likely contribute to beneficial physiological processes in the short-term (Caley et al. 2015). However, if senescent cells persist in tissues and they continue to secrete these proteases, they likely contribute to pathological processes, including impaired wound healing (Mulder and Vande Berg 2002). One of differences between acute and chronic wound environments appears to be an elevation in protease production in chronic wounds (McCarty and Percival 2013) suggesting an age-related accumulation of senescent cells in tissues could impair the wound response commonly observed in later life.

## Cellular senescence as a driver of metabolic diseases

Senescent cells have been shown to play a role in the development and progression of numerous diseases and obesity and type 2 diabetes are not an exception. Before discussing the potential role of these metabolic diseases as drivers of cell senescence, a brief overview of cell senescence as a mechanism promoting metabolic diseases is warranted.

### Cellular senescence in adipose tissue

Obesity is the predominant risk factor for development of type-2 diabetes. This suggests that biological changes associated with obesity drive the development of type-2 diabetes. Obesity is linked with an increase in the adipose tissue mass, consisting primarily of adipocytes which specialise in storing energy as fat. There are a number of studies suggesting senescent cells in adipose tissue plays a role in the development of insulin resistance and diabetes.

For example, it was found that excessive calorie intake promoted oxidative stress in the adipose tissue of mice with features of type-2 diabetes and the appearance of senescent markers such as p53, increased senescence-associated beta galactosidase activity (SA-β-Gal) and pro-inflammatory cytokines (Minamino et al. 2009). Furthermore, the upregulation of p53 in adipose tissue was shown to promote insulin resistance, whereas p53 inhibition reduced senescent markers and improved insulin resistance. In addition, markers associated with cellular senescence were also observed to be elevated in adipose tissue obtained from individuals with diabetes. In another study using a mouse model to generate elevated DNA damage and cellular senescence in adipose tissue, Chen et al. 2015 demonstrated that such mice develop obesity and glucose intolerance. p53 inhibition using pifithrin-α lead to a reduction in adipocyte senescence and reduced metabolic abnormalities. Interestingly, Monickaraj et al. (2013) reported that senescent adipocytes appear to decrease the levels of adiponectin, an adipocyte specific protein involved in the metabolism of lipids and glucose. These cells also demonstrated reduced glucose uptake, likely a response of reduced adiponectin levels.

Another mechanism by which senescent cells can promote biological decline in adipose tissue is by preventing adipogenic differentiation. It has been demonstrated that senescent adipose-derived stromal/progenitor cells in response to an adipogenic hormone cocktail show reduced expression of key adipogenic regulators and impaired expression of adipogenic differentiation genes, including adiponectin (Mitterberger et al. 2014). In another study, Zhao and Chen investigated whether elevated lipopolysaccharides (LPS) which has been associated with obesity, contribute to induction of cellular senescence and impaired adipogenic capacity (Zhao and Chen 2015). Stromal-vascular cells isolated from adipose tissue of mice subjected to LPS underwent induction of cellular senescence and a decrease in expression of adipocyte marker genes. Inhibiting LPS-induced NF-kB activation, a known regulator of the senescent secretome, did not rescue the impaired adipogenesis. More recently, Xu et al. (2015a) found that senescent human fat progenitor cells secrete activin A which can directly inhibit adipogenesis in non-senescent progenitors. Clearance of senescent cells using naturally aged INK-ATTAC mice reduced circulating activin A and enhanced adipogenesis. Collectively, these studies suggest that senescent cells within adipose tissue may promote the development of obesity, diabetes and age-related dysfunction.

### Cell senescence in pancreatic beta cells

In addition to senescent cells in adipose tissue, a limited number of studies have focused on the potential role of the senescent state in pancreatic beta cells. Since pancreatic beta cells play an essential role in insulin production, a dysfunctional response in this cell type unsurprisingly has been contributed to the pathogenesis of diabetes. Pancreatic cell dysfunction leading to impaired insulin secretion has been associated with a variety of factors including, but not limited to, increased inflammation, autoimmunity, oxidative stress and ER stress (Cerf 2013). With the current understanding of the senescent response as more than simply irreversible proliferative arrest, many of these contributing factors can be associated with induction of cellular senescence, although it would be unwise to assume that this is the only mechanism. Senescent cells are known to be pro-inflammatory, induce an immune response (likely resembling autoimmunity), display elevated levels of ROS (possibly a response to metabolic changes and mitochondria dysfunction) and in a number of cases, ER stress has been reported (Burton and Faragher 2015).

One of the first studies to suggest that senescent pancreatic beta cells may play a role in the pathogenesis of type-2 diabetes was a study undertaken by Sone and Kagawa using high-fat diet induced diabetic mice (Sone and Kagawa 2005). Following 12 months of high-fat diet, the proliferating fraction of beta cells determined by Ki67 staining was reduced to one-third compared to the control group in conjunction with an elevation (4.7-fold) in SA-beta-Gal positive cell staining. Because these changes were associated with a decrease in insulin production, it was speculated that cellular senescence contributes to the pathogenesis of diet-induced diabetes. Krishnamurthy went on to demonstrate that the senescence effector p16^INK4A^ is elevated in pancreatic islets during normal ageing in mice (Krishnamurthy et al. 2006). Furthermore, the overexpression of p16^INK4A^ comparable to normally aged mice using transgenic mice decreased islet proliferation, consequently impairing the regenerative capacity of beta cells in response to a specific beta cell toxin.

Recently, the forced induction of proliferative arrest in pancreatic beta cells via experimental overexpression of p16^INK4A^ was shown to enhance insulin secretion which improved glucose homeostasis in diabetic mice (Helman et al. 2016). However, cellular senescence is more complex than irreversible proliferative arrest alone. Through experimentally evoking a single senescent phenotype (cell-cycle arrest) as was undertaken in this instance, there is a risk of generating a “non-physiological” response leading to misleading interpretations. Since the DDR likely regulates many aspects of the senescent phenotype, including the pro-inflammatory secretome and the upregulation of immune ligands, in the absence of DNA damage, many aspects of the senescent phenotype that may normally promote a dysfunctional cell may not be presented. It is plausible that proliferative arrest alone allows pancreatic beta cells to function normally, albeit proliferation, causing these cells to grow in size. In response to an increase in cell size typical of senescent cells, all aspects of cellular physiology will also likely increase as a compensatory mechanism and this would include insulin secretion. As such, the authors advise caution when interpreting data from “senescent” models that utilise the overexpression of cyclin dependent kinase inhibitors such as p16^INK4A^ and p21^WAF-1^. Whether this response occurs naturally in vivo has yet to be determined and more research is required to further evaluate the findings of this study.

Despite the potential involvement of senescent pancreatic beta cells in diabetes, little in-depth research has been undertaken to investigate the mechanistic relationship between senescent cells and diabetes. Future advances focused on specifically eliminating senescent cells from tissues will allow researchers to directly assess the involvement of senescent cells in the pathogenesis in diabetes.

## Metabolic diseases as inducers of cell senescence

**Fig. 2:**
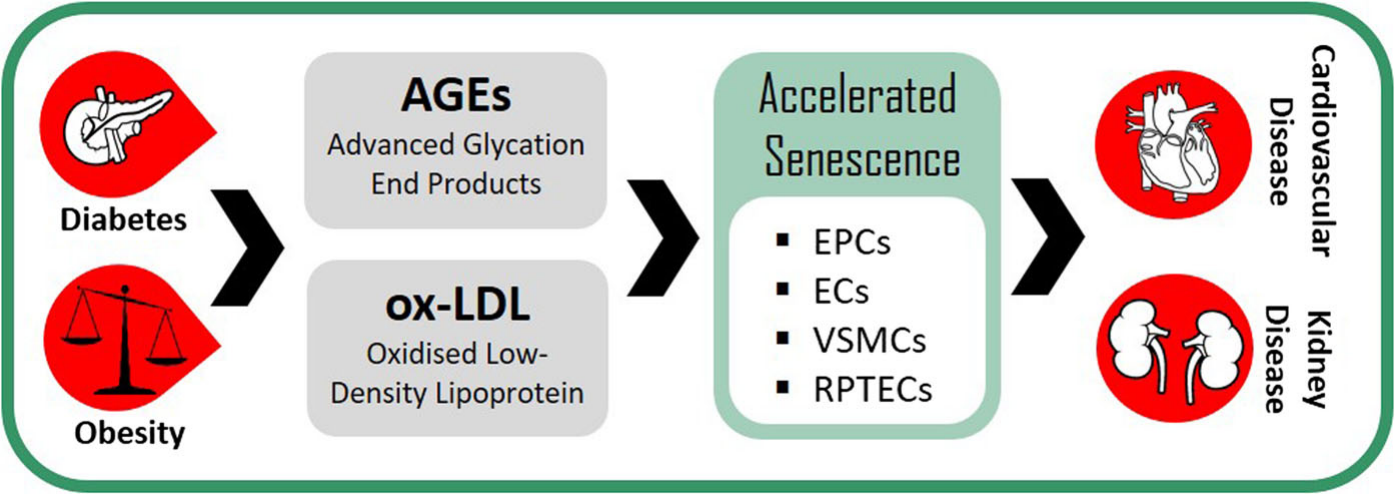
Metabolic diseases accelerate the formation of senescent cells in other tissues

Metabolic diseases such as obesity and diabetes accelerate biological ageing (Bonomini et al. 2015; Horvath et al. 2014; Rana et al. 2014; Mooradian 1988; Ronan et al. 2016; Yang et al. 2009) and consequently the appearance of age-related diseases, notably, cardiovascular disease (CVD) and kidney disease (Grundy et al. 1999; Reidy et al. 2014). One mechanism by which the ageing process could be accelerated is through premature induction of the senescent state (Fig. 2).

### Cardiovascular disease (CVD)

#### Endothelial progenitor cells

The repair of damage to the endothelial is essential in preventing the formation of atherosclerosis. Endothelial progenitor cells (EPCs) are immobilised from the bone marrow into the peripheral blood in response to vascular damage via the release of growth factors (of which VEGF appears to be an important factor), cytokines and chemokines (Young et al. 2007). However, the number and function of EPCs have been shown to be negatively correlated with various atherosclerotic risk factors and may be a consequence of accelerated senescence in EPCs (Yang et al. 2008). In particular, hypertension, often a consequence of obesity, may accelerate EPC senescence. Imanishi et al. (2005) have shown that the levels of SA-β-Gal positive EPCs increases and telomerase activity decreases in patients with hypertension and that induction of cell senescence was due to angiotensin II mediated oxidative stress. Zhou et al. (2010) later demonstrated that EPC senescence during hypertension may also be related to a reduction in calcitonin gene-related peptide (CGRP). Whilst these studies are limited and do not fully characterise the senescent state in EPCs, they do provide a basis to explore the connection between hypertension and EPC senescence in further depth. Although the mechanisms promoting EPC senescence during hypertension is unknown, it can be speculated that high blood pressure increases damage and loss of cells from the vasculature, resulting in an increase turnover of EPCs and consequently induction of replicative senescence.

The presence of diabetes is also a major risk factor in the development of CVD (Fadini et al. 2005) and may also accelerate the appearance of senescent EPCs. Diabetes is associated with high blood glucose levels which may contribute to premature cell senescence. Kuki et al. (2006) demonstrated that EPCs cultured under high glucose levels undergo accelerated senescence. Chen et al. (2007) also demonstrated that high glucose induces a senescent state in EPCs associated with a decrease in nitic oxide synthase activity. Furthermore, this study also demonstrated that EPC migration in response to VEGF was impaired in high glucose-induced senescent EPCs, suggesting a potential mechanism by which induction of the senescent state impairs EPC function.

The presence of elevated oxidised low density lipoproteins (ox-LDL) observed in diabetics has also been shown to reduce the number and impair function of circulating EPC. Providing further support for the link between diabetes and EPC senescence, Imanishi et al. (2004) demonstrated that ox-LDL can induce EPC senescence leading to EPC dysfunction. A further study supports these findings, reporting that EPCs from normal donors cultured in the presence of ox-LDL underwent cell senescence associated with Akt activation and that EPCs from diabetic patients showed a similar phenotype to these ox-LDL induced senescent EPCs (Rosso et al. 2006). Although senescence was not specifically mentioned and instead referred to as EPC dysfunction, Ji et al. (2015) showed that ox-LDL decreased the proliferation, migration and adhesion capacity of EPC. In addition, ox-LDL treated EPCs displayed increased ROS production, NADPH oxidase expression and activation of the NF-kB pathway, a known regulator of the pro-inflammatory secretome of senescent cells. An increase in NADPH oxidase has also been reported in ox-LDL induced cell senescence in macrophages (Wang et al. 2018).

#### Vascular endothelial cells

Vascular endothelial cells (EC) play an important role in regulating vascular tone (via nitric oxide activity), cellular adhesion and filtration, smooth muscle cell proliferation and thromboresistance (resistance to non-haemostatic blood clotting). As such, when endothelial cells become dysfunctional, the biological consequence can be multifaceted. Induction of cellular senescence is likely one mechanism promoting endothelial dysfunction.

High glucose levels associated with diabetes have also been shown to promote EC senescence in vitro. Culturing HUVECs under high glucose was shown to induce HUVEC senescence and is again associated with a reduction in NO synthesis (Zhong et al. 2010). In this instance, co-incubation with l-arginine (a substrate for NO synthesis) reduced the appearance of senescent-like cells and increased NO production, suggesting that administration of l-arginine could improve vascular function during CVD. Arginase 1 is an enzyme which catalyses the hydrolysis of arginine to urea and ornithine. A recent study showed that arginase 1 gene deletion or pharmacology inhibition protected against induction of EC senescence in diabetic mice (Shosha et al. 2018). Conversely, arginase 1 overexpression or high glucose treatment increased SA-β-Gal activity in cultured ECs, suggesting that inhibition of arginase activity may be a strategy for preventing premature senescence.

Another study demonstrated that high glucose-induced EC senescence could be reduced through the use of the drug Donepezil via SIRT1 activity (Zhang et al. 2015). Interestingly, it appears that intermitted high glucose rather than constant high glucose is more likely to promote EC senescence (Maeda et al. 2015). Intermitted high glucose induced EC senescence involved the upregulated expression of p22^phox^, an NADPH oxidase component, increasing superoxide. Another study investigated the impact of insulin on glucose-induced endothelial senescence in HUVECs and human aortic endothelial cells, demonstrating that physiological concentrations of insulin could delay the appearance of senescent-like ECs under high glucose conditions, associated with a reduction in ROS generation and an increase in NO (Matsui-Hirai et al. 2011).

High blood glucose levels can also lead to the glycation of proteins, consequently disrupting their normal function. In addition, advanced glycation end-products (AGEs) may interact with membrane receptors of cells that alter intracellular signalling and promoting the production of pro-inflammatory cytokines and ROS (Singh et al. 2014). There have been a limited number of studies which suggest that glycation products may act as a trigger for inducing cell senescence. For example, culturing early-passage endothelial cells on glycated collagen was reported to increase premature senescence associated with a decrease in nitric oxide (NO) production (Chen et al. 2002). Furthermore, SA-β-Gal activity was elevated in ECs of aortas derived from Zucker diabetic rats compared to control rats. Interestingly, obesity is known to impair vasodilation of blood vessels and therefore blood flow (a CDV risk factor) by decreasing NO production (Toda and Okamura 2013), suggesting that obesity accelerated induction of senescent ECs may contribute to this impairment. A number of other studies have suggested that advanced AGEs play a role in “endothelial dysfunction” although not necessarily related to senescence induction (Goldin et al. 2006).

As with EPCs, there are also some indications to suggest that ox-LDL may also accelerate EC senescence. Rats fed on a high fat diet to establish a hyperlipidemic model, resulted in increased plasma lipids, endothelium-derived myeloperoxidase (MPO) expression, EC senescence and endothelial dysfunction associated with a reduction in glycogen synthase 3 beta (GSK-3β) activity and p-β-catenin (Liu et al. 2015b). In vitro treatment of ECs with ox-LDL also induced MPO expression and EC senescence, associated with a decrease in GSK-3β and p-β-catenin, thus supporting their in vivo findings. Inhibition of MPO reduced the number of senescent cells in vitro, suggesting senescence induction in this instance is partially mediated via the production of MPO-derived hypochlorous acid (HOCl).

#### Vascular smooth muscle cells

Vascular calcification, referring to the deposition of calcium phosphate mineral in the intima or media of arterial walls is a major risk factor in CVD as it leads to reduced elasticity and compliance of arteries (Wexler et al. 1996). The presence of diabetes is known to accelerate arterial calcification, in which VSMCs appear to transform into osteoblast-like cells, thought to be mediated in part by high glucose levels (Chen and Moe 2003; Tyson et al. 2003). Interestingly, a number of studies have reported that VSMCs undergoing cell senescence adopt a pro-calcificatory phenotype. Microarray analysis of replicative senescent VSMCs suggested that senescent VSMCs adopt a pro-calcificatory phenotype associated with the repression of matrix Gla protein (MGP), an inhibitor of calcification and the up-regulation of bone morphogenetic protein (BMP2), which promotes calcification (Burton et al. 2009). These findings were supported by Nakano-Kurimoto et al. (2009), who demonstrated that replicative senescent VSMCs indeed undergo calcification as determined by Alizarin red staining, associated with increased expression of alkaline phosphatase via runt-related transcription factor-2 (RUNX-2). It thus appears that senescent VSMCs adopt a cell type specific senescent phenotype associated with calcification (Burton et al. 2010).

Whilst there does not appear to be any studies investigating whether AGEs can induce senescence in VSMCs, there are a number of reports demonstrating that AGEs induce calcification in VSMCs. Wang et al. demonstrated that AGEs could promote human aortic smooth muscle cell (HASMC) calcification through activating NF-kB and downregulating IGF1R (Wang et al. 2013). Adding further insight into a possible mechanism of VSMC calcification, Wei et al. (2013) showed that AGE-induced calcification in VSMC is associated with enhanced Nox1 expression and consequently the production of intracellular superoxide anions. Although senescent cells are often associated with NF-kB activation and elevated ROS, it cannot be assumed that induction of the senescent state is the primary mechanism promoting calcification in these instances. Further work is required to fully evaluate the possible link between AGEs, calcification and cell senescence.

### Kidney disease

High glucose levels associated with diabetes can result in kidney damage (diabetic nephropathy) leading to the build-up of waste fluids in the blood (Shahbazian and Rezaii 2013). As with CVD, there is some evidence suggesting that kidney damage may partially be due to senescence induction. Verzola et al. (2008) investigated whether cellular senescence could be a mechanism promoting kidney damage in type 2 diabetic nephropathy (DN). Renal biopsies from patients with type 2 DN showed a three-fold increase in SA-β-Gal activity within the tubular compartment of diabetic kidneys and an increase in p16 expression in both tubules and podocytes compared to controls. Furthermore, proximal tubule cells cultured under high glucose reduced the proliferative capacity of cells, increasing both SA-β-Gal staining and p16 indicative of cell senescence. Kitada et al. (2014) later demonstrated using a type-1 diabetic mouse model, that hyperglycaemia increases renal expression of p21 and SA-β-Gal staining in tubular epithelial cells in the early stages of diabetic nephropathy. Again, cultured human proximal tubular cells in high glucose elevated the expression of senescence markers, whilst knockdown of p21 or sodium glucose cotransporter (SGLT) 2 prevented senescence induction.

As with cardiovascular related cells, AGEs have also been shown to induce premature senescence in proximal tubular epithelial cells via activation of endoplasmic reticulum stress (Liu et al. 2015a). Blocking receptors for advanced glycation end-products (RAGE) reduced AGE-induced premature senescence. In another study, Ikeda et al. (2011) investigated the effects of AGEs in primary renal proximal tubular epithelial cell (RPTECs) senescence by modulating glyoxalase I (GLO1) which detoxifies precursors (i.e. glycoxal) of AGEs. Aged GLO1 overexpressing transgenic rats showed reduced levels of cell senescence associated with reduced accumulation of renal AGEs. Furthermore, GLO1-transgenic rats had reduced age-related interstitial thickening and were protected against age-dependent decline in renal function. Validating these in vivo findings in vitro, late passage renal proximal epithelial cells showed elevated senescence markers that were reduced in cells overexpressing GLO1 or accelerated through GLO1 knockdown. Glyoxal has also been shown to induce cell senescence in fibroblasts and mesenchymal stem cells (Larsen et al. 2012; Sejersen and Rattan 2007).

It thus appears that diabetes may promote kidney disease by accelerating kidney ageing through premature induction of cell senescence. A number of studies have looked at the possible link between cellular senescence and kidney function decline associated with ageing. Berkenkamp et al. (2014) investigated age-related changes in tubular epithelial cells in young and aged mice and in vitro. Kidneys from aged mice showed more senescence markers (SA-β-Gal, p16, γH2AX, Ki67) and an elevation in cyclin D1 compared to young mice. Although cyclin D1 is commonly considered as a proliferation marker which was expected to decline with age in this instance, cyclin D1 has been reported as a marker of cell senescence, possibly functioning to inhibit p21 degradation (Burton et al. 2007; Coleman et al. 2003). In another age-related study focused on rat kidneys, Melk et al. (2003) also reported that aged rats (9–24 months) also show a 72-fold increase in p16^INK4A^ RNA expression, an increase in SA-β-Gal staining within the epithelium, but without a significant change in telomere length.

### Cancer

Obesity is a well-known risk factor for the development of a variety of different cancers, likely owing in part to increased secretion of hormones into the bloodstream leading to functional dysregulation in recipient cells (Basen-Engquist and Chang 2011). However, one interesting study has provided evidence which suggests that obesity induces altered gut microbial metabolites which can promote liver cancer through cell senescence induction (Yoshimoto et al. 2013). Induction of obesity in mice altered the gut microbiota leading to an elevation in the levels of the gut bacterial metabolite, deoxycholic acid (DCA), known to induce DNA damage. DCA was shown to induce hepatic stellate cell (HSC) senescence associated with a pro-inflammatory tumour promoting secretome that facilitated hepatocellular carcinoma (HCC) development in the livers. Blocking DCA production, reducing gut bacteria or inhibiting the senescent secretome (IL1β absence) all prevented HCC development. Research focused on obesity induced cellular senescence and its role in promoting cancer is at its infancy and it will interesting to see what further developments are made within this field.

## Therapeutic targeting of senescent cells

Senescent cells likely play a role in the development and progression of metabolic diseases such as obesity and diabetes, which in turn can lead to diabetes-induced senescence and the formation of secondary diseases such as CVD and kidney disease. Therefore, therapeutically targeting senescent cells has great potential for improving health (Gurău et al. 2018). Such strategies may include (1) senescent cell prevention, (2) senescent cell reversal, (3) inhibiting aspects of the senescent phenotype and (4) senescent cell elimination.

Inhibiting molecular pathways important for induction into cell senescence could prevent cells under certain conditions (such as high glucose) from becoming senescent. As mentioned above with regard to EC senescence, this may include the use of drugs which inhibit arginase 1 and SIRT1 activity. In addition to preventing cell senescence, it may one day be possible to reverse cell senescence. It has been suggested that the use of resveratrol-related compounds (resveralogues) can rescue cells from senescence, but further research is required (Latorre et al. 2017). The third therapeutic approach is the inhibition of the damaging components of the senescent phenotype, primarily the pro-inflammatory secretome. The regulation of the senescent secretome has been research extensively in recent years and an understanding of the molecular pathways involved has enabled its inhibition via drugs (reviewed in Malaquin et al. 2016). As an example of the potential benefits, findings from one study suggested that inhibition of senescent secretome in mice reduces frailty in old age (Xu et al. 2015b). The elimination of senescent cells by induction of cell death has provided the most convincing evidence in mice that senescent cells play a role in diseases and that their removal can be beneficial to health (reviewed in McHugh and Gil 2018). As such, there have been several drugs in recent years that have been identified or created which can specifically kill senescent cells (Childs et al. 2017; de Keizer 2017; Muñoz-Espín et al. 2018; Soto-Gamez and Demaria 2017). In addition to the use of pharmacological drugs, it may be possible to use our own immune system to target the removal of senescent cells in a similar manner that immunotherapy is now being used to target some cancers (Burton and Stolzing 2018).

Currently the use of drugs is justified only when a disease has manifested and is problematic. In addition, there are often specific drugs for specific diseases. If many different diseases have a common underlying cause, such as cell senescence, it may one day be possible to treat many different diseases with a single drug. However, rather than waiting for a disease to present itself, intermittent removal of senescent cells throughout our lifetime could be utilised as a preventative measure, ensuring long and healthy lives.
